# The Preparation of Teeth for Filling

**Published:** 1867-12

**Authors:** A. P. Southwick


### THE PREPARATION OF TEETH FOR FILLING.

BY DR. A. P. SOUTHWICK.

Read before the Western New York Dental Society.

Mr. President and Gentlemen: I shall not attempt to make any apologies for the shortness and imperfections of this paper, as it was not written until the last moment; but having promised to write something, however short, I thought you would accept a poor paper rather than have me stay at home, which I otherwise should have done.

I regret that the subject on which I am writing (the preparation of teeth for filling) had not fallen into more able hands that could have done it justice; for the subject is full of interest, especially to the young practitioner. For his success in the treatment of caries of the teeth is entirely dependent upon his knowledge and ability to so manipulate as to produce the best results. His teachings should be from the best authority, that he may commence aright; and all his operations should be governed by some fixed rules.

As the limits of this paper and the ability of the writer will not admit of any amount of spread eagle, I shall direct my remarks to the younger members of this association, It is the difficulties encountered in every day practice that most concern them, and wishing to lend a helping hand to the worthy seeking information, I shall give in the plainest and shortest manner possible a few ideas in the preparation of the cavities most generally found in the teeth.

And in order that we may classify them somewhat, and you follow me the more understandingly, I will commence with the central incisors, giving each tooth some little attention.

In preparing this class of teeth it is very essential that you control the time of your patient as far as may be necessary. You cannot operate without sufficient space, and this can be got in no proper way except by pressure; this can be produced by rubber drawn between the teeth, or what is far better and pleasanter for the patient, packing a small pellet of cotton firmly between the teeth changing it daily, and in from two to four days in most cases, you will have obtained sufficient room.

On removing the cotton the first thing to be done is to place a wedge made of some dense wood, firmly between the teeth above the cavity, this is an important point, and must be done in all cases of approximal decay, this keeps the tooth firm, and also makes a dam excluding the moisture from the cavity; remove with an excavator the soft caries, then with a small chisel trim the margin of the cavity until you have a firm wall both upon the outer and inner surface, beveling slightly from the cavity, that the filling when completed may support the tooth.

With a hoe shaped excavator or the point of a chisel, clean the cervical wall; this is the most essential part of the cavity, for here the enamel is very thin or may be entirely gone, and is the point most likely to be attacked the second time, and in fact is the point where most fillings fail. Then with a narrow pointed hoe or diamond point cut a small groove around and as near the bottom of the cavity as possible; then with a small drill make two pits or retaining points in the cervical wall, and one at the cutting edge, and your work is done. Since the introduction of the Morgan

& Lamm Gold, these retaining points are not essential if the whole cavity is retentive in form.

The cuspid teeth can be prepared similar to the centrals, if the enamel is sufficiently firm; if not, the best way is to cut through the crown or palatial surface, making a compound filling when completed.

In the preparation of cavities in the bicuspid, it should be done with the utmost care; the decay is almost universally approximal and deep seated, extending often under the margin of the gum and beyond the enamel.

In most cases it will be necessary to separate these teeth with a file, sometimes with a V shape, if the teeth are much crowded. The crowns of these should be cut through, making the passage through nearly as wide as the decayed portion.

Great care must be exercised in thoroughly cleansing the cervical wall, and nicely rounding the edges of the cavity along the proximal surfaces. I consider the bicuspids the most difficult teeth in the mouth to save when once the decay has a good start, and with the nicest manipulating and the utmost care they are frequently lost.

The approximal cavities in the molars should be treated much the same as the bicuspids, cutting boldly through the crown, that you may more thoroughly reach the bottom both in excavating and in the filling. A few words in regard to the preparation of cavities in the grinding surfaces, commonly called crown cavities, and I have done, although imperfectly, with this part of my subject.

Chisels should be used almost exclusively in opening these cavities and following up the seams to their end, being careful to leave no angles that cannot be filled perfectly; the preparation is very simple, but must be done thoroughly to insure success.

The remaining part of my subject, reasons for annealing gold, is a subject of considerable importance, perhaps more than most of us suspect.

By the constant changes of temperature all bodies are surrounded with a film of moisture, constantly absorbing and throwing off, making it impossible for any two separate bodies to come in perfect contact so long as this state of things exist. By subjecting gold to a moderate heat, this moisture is driven off, and the gold having an affinity for itself, readily unites. This theory is often proved in cases where a patient with a moist breath will entirely destroy a filling, by being allowed to breathe upon it while in the process of manipulating, the gold in the tooth being colder than the breath it absorbs the heat, and surrounding itself with the condensed moisture, thereby destroying its adhesiveness.

In the preparation of gold for filling, we are obliged to handle it, thereby coating it with the perspiration from the hands, which is still more detrimental to the adhesiveness than the moisture from the air; making it all the more necessary to anneal thoroughly. And to insure perfect unity and consequent success, the gold should be kept hot during the process of filling, and worked as rapidly as possible.

